# Hospital Needs

**Published:** 1912-09-21

**Authors:** 


### Hospital Needs.

Krüschen Salts have been much before the public during the last year or two, and their merits are in consequence being appraised by an increasing proportion of the population. There is little doubt that town-dwellers engaged in sedentary occupations—or in none at all—are especially liable to suffer from defective elimination and from constipation, and that these errors are at the root of many diverse ailments and diseases. It is also true that many people cannot overcome this tendency except by the use, habitual or occasional, of aperient medicines, among which salines have an old-established and well-deserved pre-eminence. Krüschen Salts contain, according to analysis, sulphate of soda, chloride of soda, phosphate of soda, sulphate of potash, and chloride of potash. They are not unpleasant to take, are effective in action, and are a preparation of undoubted purity and reliability.

